# Myeloid differentiation primary response gene 88-leukotriene B4 receptor 2 cascade mediates lipopolysaccharide-potentiated invasiveness of breast cancer cells

**DOI:** 10.18632/oncotarget.3304

**Published:** 2015-01-21

**Authors:** Geun-Soo Park, Jae-Hong Kim

**Affiliations:** ^1^ College of Life Sciences and Biotechnology, Korea University, Seoul, Korea

**Keywords:** LPS, MyD88, Invasiveness, BLT2, IL-6/IL-8

## Abstract

Inflammation and local inflammatory mediators are inextricably linked to tumor progression through complex pathways in the tumor microenvironment. Lipopolysaccharide (LPS) exposure to tumor cells has been suggested to promote tumor invasiveness and metastasis. However, the detailed signaling mechanism involved has not been elucidated. In this study, we showed that LPS upregulated the expression of leukotriene B_4_ receptor-2 (BLT2) and the synthesis of BLT2 ligands in MDA-MB-231 and MDA-MB-435 breast cancer cells, thereby promoting invasiveness. BLT2 depletion with siRNA clearly attenuated LPS-induced invasiveness. In addition, we demonstrated that myeloid differentiation primary response gene 88 (MyD88) lies upstream of BLT2 in LPS-potentiated invasiveness and that this ‘MyD88-BLT2’ cascade mediates activation of NF-κB and the synthesis of IL-6 and IL-8, which are critical for the invasiveness and aggression of breast cancer cells. LPS-driven metastasis of MDA-MB-231 cells was also markedly suppressed by the inhibition of BLT2. Together, our results demonstrate, for the first time, that LPS potentiates the invasiveness and metastasis of breast cancer cells via a ‘MyD88-BLT2’-linked signaling cascade.

## INTRODUCTION

Lipopolysaccharide (LPS) is a key component of the outer membrane of Gram-negative bacteria and is specifically recognized by Toll-like receptor 4 (TLR4) [1, 2]. Recent studies have demonstrated that TLR4 is expressed in a wide variety of tumors, including breast cancers, and that LPS-TLR4 signaling promotes cancer progression [3-7]. For example, LPS has been shown to increase invasiveness in various cancer cells, and silencing TLR4 reduced their metastatic potential [8-10]. Whereas the role of LPS-TLR4 in potentiating tumor invasion and metastasis has been defined, the detailed signaling mechanism responsible for this involvement remains largely unknown.

Leukotriene B_4_ (LTB_4_) is a potent chemoattractant and a local proinflammatory lipid mediator that plays a role in innate immunity [11]. Recent studies have suggested that LTB_4_ and its receptor BLT2 are associated with tumor progression [12-17]. An increased abundance of LTB_4_ and BLT2 has been observed in many types of tumors, including neuroblastomas, as well as pancreatic, bladder, breast and ovarian cancers [12-14, 18, 19]. In addition, a BLT2 inhibition reduced the incidence of metastasis in an *in vivo* mouse model [19-21]. Despite of these potential properties of BLT2 as a pro-tumorigenic mediator, its role in LPS-driven cancer potentiation has not been reported yet.

In this study, we found that LPS upregulated the expression of BLT2 in MDA-MB-231 and MDA-MB-435 cell lines, thereby increasing the invasive potential of these aggressive breast cancer cells. In addition, we showed that MyD88 functions upstream and that NF-κB functions downstream of BLT2. We also showed that IL-6 and IL-8 lie downstream of BLT2-NF-κB in the LPS cascade potentiating invasiveness. Together, our results describe a novel LPS-induced ‘MyD88-BLT2-NF-κB-IL-6/IL-8’ signaling cascade that promotes breast cancer progression. Our findings thus provide novel insight into how LPS potentiates the invasiveness and metastasis of breast cancer cells.

## RESULTS

### LPS enhances the invasive potential and the level of BLT2 expression in MDA-MB-231 and MDA-MB-435 cells

**Figure 1:**
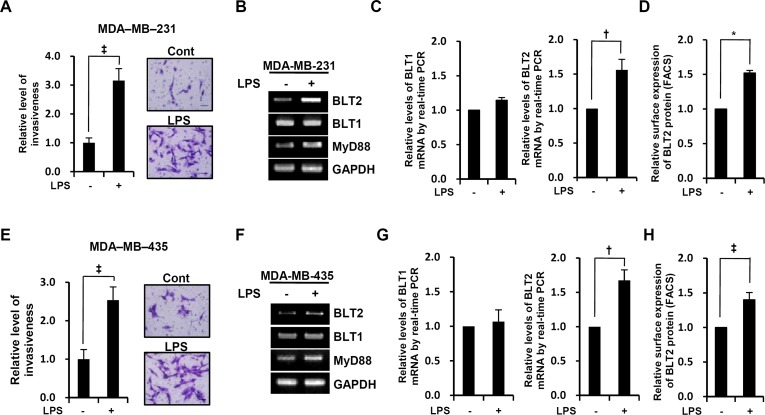
LPS enhances the invasive potential and BLT2 expression in MDA-MB-231 and MDA-MB-435 cells MDA-MB-231 cells (A) were treated with LPS (0, 1 μg/ml) for 24 h and MDA-MB-435 cells (E) were treated with LPS (0, 1 μg/ml) for 48 h and then assayed for invasiveness. Representative fields of invading cells stained with hematoxylin and eosin (H&E) were photographed with a CKX41 microscope equipped with a DP71 digital camera (Olympus) at 40X magnification (Scale bars, 50 μm). Quantitative data are expressed relative to the values for cells treated with PBS control. MDA-MB-231 and MDA-MB-435 cells were treated with LPS (0, 1 μg/ml) for 24 h, after which mRNA levels of MyD88, BLT1, and BLT2 were measured by semiquantitative RT-PCR (B and F). In addition, cells treated with LPS (0, 1 μg/ml) for 24 h were subjected to either quantitative real-time PCR for BLT1 (*left* panel) and BLT2 (*right* panel) (C and G) or FACS analysis for BLT2 protein levels (D and H). The semiquantitative RT-PCR data are representative of three independent experiments, and all quantitative data are shown as the mean ± SD of three independent experiments. ^*^p < 0.05, ^†^*p* < 0.01, ^‡^*p* < 0.005.

We assessed whether LPS could enhance the invasive potential of MDA-MB-231 and MDA-MB-435 cells. Their invasiveness was significantly increased by exposure to LPS (Fig. 1A and E). To understand the signaling mechanism by which LPS enhances the invasive potential of these breast cancer cells, we examined whether LPS upregulated BLT2 mRNA. Both semiquantitative RT-PCR (Fig. 1B and F) and quantitative real-time PCR analysis (Fig. 1C and G) revealed that the amount of BLT2 mRNA was indeed markedly increased by LPS treatment, whereas BLT1 expression was not affected. BLT2 protein levels, as determined by flow cytometry, were also increased by LPS (Fig. 1D and H). In agreement with previous reports, LPS also increased MyD88 expression in these cells [9, 25] (Fig. 1B and F).

### BLT2 inhibition attenuates the invasive potential of MDA-MB-231 cells

**Figure 2:**
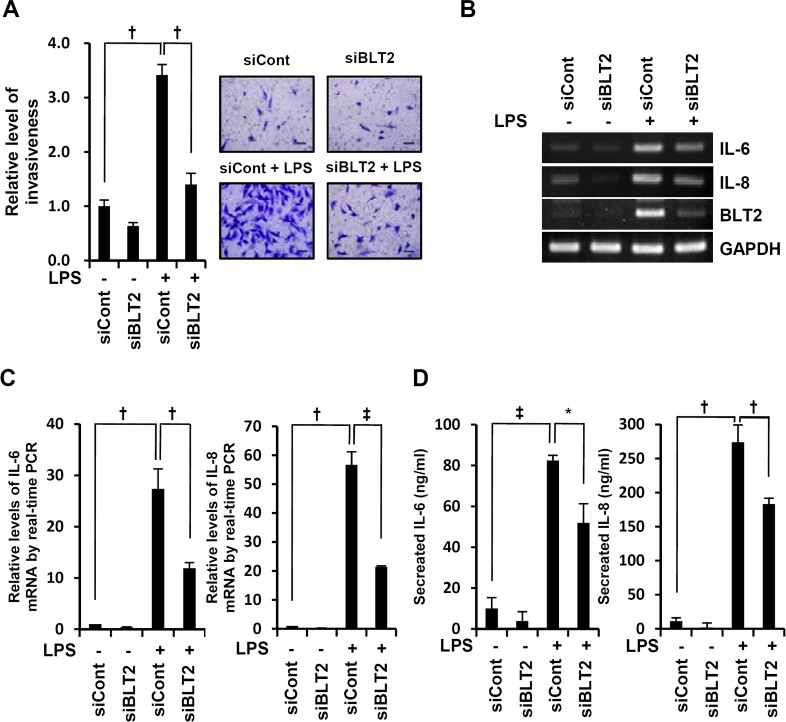
BLT2 inhibition attenuates the invasive potential of MDA-MB-231 cells (A) MDA-MB-231 cells were transfected with BLT2 (siBLT2) or control (siCont) siRNA for 24 h and then assayed for invasiveness in the presence or absence of LPS (1 μg/ml). Scale bars, 50 μm. (B and C) Cells were treated as in (A) and then assayed for IL-6, IL-8, and BLT2 mRNA levels by semiquantitative RT-PCR (B) and quantitative real-time PCR for IL-6 (*C, left* panel) and IL-8 (C, *right* panel). (D) Cells were treated as in (A), and the amount of secreted IL-6 (*left* panel) and IL-8 (*right* panel) in culture supernatants was determined by ELISA. The semiquantitative RT-PCR data are representative of three independent experiments, and all quantitative data are shown as the mean ± SD of three independent experiments. ^*^p < 0.05, ^†^*p* < 0.01, ^‡^*p* < 0.005.

To investigate whether BLT2 upregulation is associated with LPS-induced invasiveness, we examined the effect of depleting BLT2 on invasion. BLT2 depletion using siRNA clearly attenuated the LPS-induced invasive activity of MDA-MB-231 cells (Fig. 2A), whereas inhibition of BLT1 had no effect in LPS-induced invasive activity (data not shown). Previous research has shown that IL-6 and IL-8 are associated with the invasiveness of breast cancer cells [19, 26]. Consistent with these reports, we observed that LPS-induced invasiveness was decreased by antisense knockdown of IL-6 and IL-8 (data not shown). Furthermore, BLT2 knockdown suppressed the LPS-induced increase in IL-6 and IL-8 (Fig. 2B, C and D). Together, these results suggest that LPS-enhanced invasiveness is through a ‘BLT2-IL-6/IL-8’-linked cascade.

### Inhibition of BLT2 ligands synthesis suppresses LPS-enhanced invasive potential and IL-6, IL-8 synthesis

**Figure 3:**
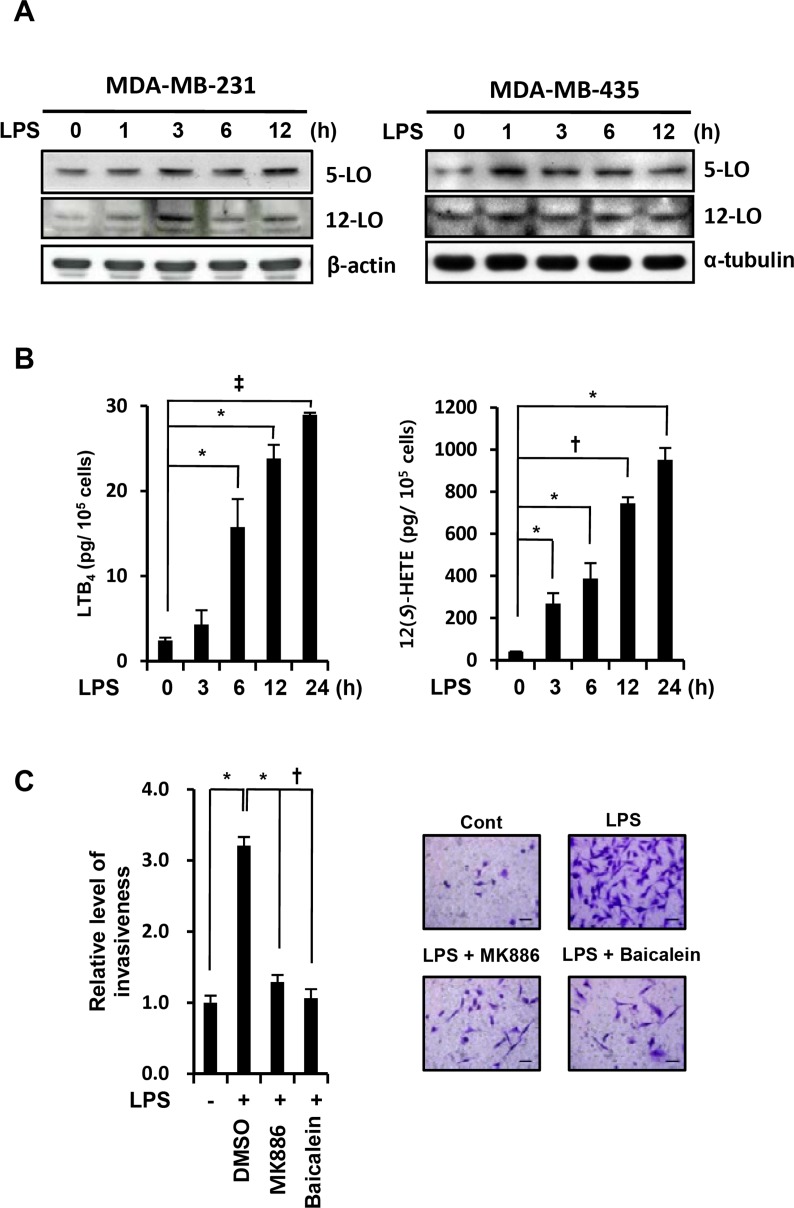
Inhibition of BLT2 ligands synthesis suppresses LPS-enhanced invasive potential and IL-6, IL-8 synthesis (A) MDA-MB-231 cells (*left* panel) and MDA-MB-435 cells (*right* panel) were treated with LPS (1 μg/ml) for the indicated times (0, 1, 3, 6, and 12 h), and then, cell lysates were subjected to immunoblot analysis with antibodies to 5-LO, 12-LO, α-tubulin (loading control), and β-actin (loading control). (B) MDA-MB-231 cells were incubated with LPS (1 μg/ml) for the indicated times (0, 3, 6, 12, and 24 h), after which, the cell supernatants were measured by ELISA. (C) MDA-MB-231 cells were incubated for 30 min with MK886 (5 μM) and baicalein (20 μM) and then for 24 h in the presence or absence of LPS (1 μg/ml), after which, they were assayed for invasiveness. Scale bars, 50 μm. Representative fields of invading cells stained with hematoxylin and eosin (H&E) are shown. All quantitative data are shown as the mean ± SD of three independent experiments. ^*^p < 0.05, ^†^*p* < 0.01, ^‡^*p* < 0.005.

Ligands for BLT2 include eicosanoids, such as LTB_4_, 12(*S*)-HETE, and 12(*S*)-hydroxyheptadeca-5Z,8E,10E-trienoic acid (12(*S*)-HTT) [27]. The biosynthesis of LTB_4_ and 12(*S*)-HETE from arachidonic acid is catalyzed by 5-lipoxygenase (5-LO) and 12-lipoxygenase (12-LO), respectively [28]. To examine whether LPS upregulates the biosynthesis of the BLT2 ligands LTB_4_ and 12(*S*)-HETE, we determined the level of these ligands and the respective lipoxygenase enzyme levels in LPS-treated MDA-MB-231 cells. The expression of 5-LO and 12-LO were increased in response to LPS stimulation in MDA-MB-231 and MDA-MB-435 cells (Fig. 3A). Also, their metabolites LTB_4_ and 12(*S*)-HETE were all markedly increased in response to LPS stimulation (Fig. 3B). LPS-induced invasion of MDA-MB-231 cells was attenuated by pretreatment with either the 5-LO activating protein (FLAP) inhibitor MK886 or the 12-LO inhibitor baicalein (Fig. 3C). Together, these results suggest that upregulation of the BLT2 ligands (LTB_4_ and 12(*S*)-HETE) is necessary for the LPS-enhanced invasive potential.

### LPS-enhanced invasive potential is through a MyD88-BLT2-linked cascade

**Figure 4:**
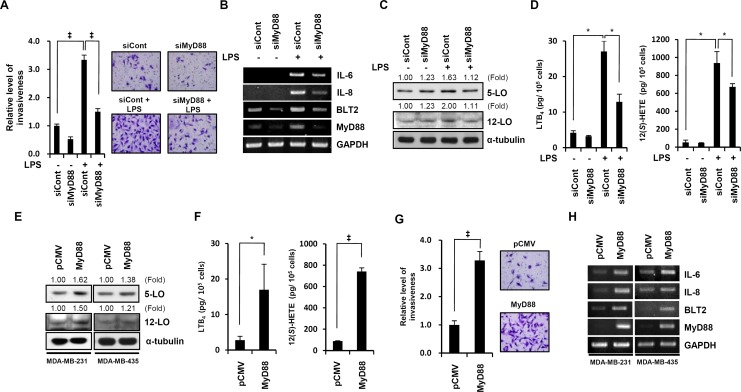
LPS-enhanced invasive potential is through a MyD88-BLT2-liked cascade (A) MDA-MB-231 cells were transfected with MyD88 (siMyD88) or control (siCont) siRNAs. After 24 h, the cells were assayed for invasiveness in the presence or absence of LPS (1 μg/ml) for 24 h. Scale bars, 50 μm. (B and C) Cells were treated as in (A) and then assayed by semiquantitative RT-PCR for IL-6, IL-8, BLT2, and MyD88 mRNA levels (B) or immunoblot to determine the protein levels of 5-LO, 12-LO, and α-tubulin (loading control) (C). (D) Cells were treated as in (A), and then, the culture supernatants were assayed for amounts of LTB_4_ (*left* panel) and 12(*S*)-HETE (*right* panel) by ELISA. (E) MDA-MB-231 cells (*left* panel) and MDA-MB-435 cells (*right* panel) were transfected with an expression plasmid for MyD88 or the empty plasmid (pCMV) and then incubated for 24 h. Then, the cell lysates were subjected to immunoblot analysis for 5-LO, 12-LO, and α-tubulin. (F) Cells were transfected as in (E), and then, the quantity of LTB_4_ and 12(*S*)-HETE in the culture supernatants was measured by ELISA. (G) Cells were transfected as in (E) and then the cells were assayed for invasiveness. Scale bars, 50 μm. (H) Cells were transfected as in (E), RNA was isolated and subjected to semiquantitative RT-PCR analysis for IL-6, IL-8, BLT2, MyD88 and GAPDH (loading control) mRNAs. Semiquantitative RT-PCR data are representative of three independent experiments, and all quantitative data are shown as the mean ± SD of three independent experiments. *p* < 0.05, ^‡^*p* < 0.005.

We next tested whether MyD88 plays a role in the upregulation of BLT2 during LPS-driven invasion. MyD88 depletion by siRNA resulted in a marked attenuation of LPS-induced invasiveness (Fig. 4A) and also suppressed the LPS-induced increases in BLT2, IL-6 and IL-8 mRNA and 5-LO and 12-LO protein in MDA-MB-231 cells (Fig. 4B and C). Next, we showed that the LPS-induced synthesis of LTB_4_ and 12(*S*)-HETE was reduced by MyD88 depletion in MDA-MB-231 cells (Fig. 4D). To further examine the relationship between MyD88 and BLT2, we examined the effects of transient transfection with a MyD88 expression plasmid. Overexpression of MyD88 increased 5-LO and 12-LO proteins and the secretion of their metabolites LTB_4_ and 12(*S*)-HETE (Fig. 4E and F). MyD88 overexpression also increased the invasiveness of MDA-MB-231 cells (Fig. 4G) and the mRNA levels of IL-6, IL-8 and BLT2 (Fig. 4H). Also, the BLT2 inhibition significantly attenuated this MyD88-induced invasion and IL-6/IL-8 synthesis (data not shown). Together, these results show that the LPS-induced increases in invasiveness and biosynthesis of IL-6 and IL-8 occur through a MyD88-BLT2-linked cascade.

### NF-κB is downstream of BLT2 in mediating the LPS-enhanced invasive potential in MDA-MB-231 cells

**Figure 5:**
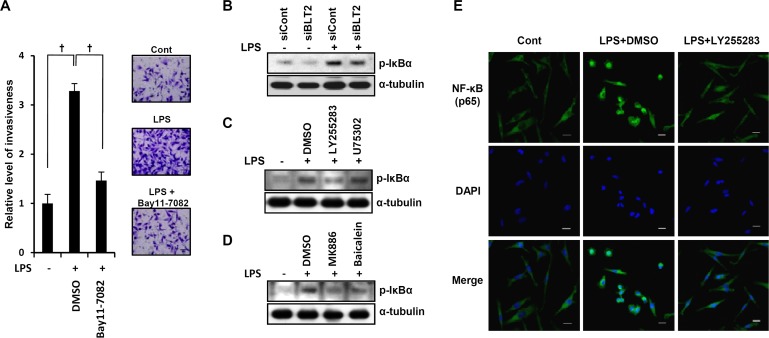
NF-κB is downstream of BLT2 in mediating the LPS-enhanced invasive potential in MDA-MB-231 cells (A) MDA-MB-231 cells were incubated for 30 min with Bay11-7082 (10 μM) or DMSO and then for 24 h in the presence or absence of LPS (1 μg/ml), after which, they were assayed for invasiveness. Scale bars, 50 μm. (B) Cells were transfected with siBLT2 or siCont, incubated for 24 h, and then stimulated with LPS (1 μg/ml) for 24 h, after which, the cell lysates were subjected to immunoblot analysis with antibodies to either phosphorylated or total IκBα. (C) Cells were incubated for 30 min with LY255283 (10 μM), U75302 (1 μM), or DMSO and then for 24 h in the presence or absence of LPS (1 μg/ml), after which, the cell lysates were subjected to immunoblot analysis as in (B). (D) Cells were incubated for 30 min with MK886 (5 μM), baicalein (20 μM), or DMSO and then for 24 h in the presence or absence of LPS (1 μg/ml), after which, the cell lysates were subjected to immunoblot analysis as in (B). (E) Cells were incubated for 30 min with LY255283 (10 μM) or DMSO, stimulated with LPS (1 μg/ml) for 24 h, and then subjected to immunofluorescence staining with antibodies to the p65 subunit of NF-κB (green). Nuclei were also stained with 4′,6-diamidino-2-phenylindole (DAPI, blue), and the cells were examined with a confocal microscope. Scale bars, 20 μm. All quantitative data are shown as the mean ± SD of three independent experiments. ^†^*p* < 0.01.

To understand the signaling cascade downstream of BLT2 involved in the LPS-enhanced invasiveness, we examined the role of NF-κB. We observed that the NF-κB inhibitor Bay 11-7082 greatly inhibited LPS-induced invasiveness (Fig. 5A). In addition, BLT2 inhibition with LY255283 or BLT2 depletion by RNAi resulted in a marked reduction of LPS-induced phosphorylation of IκBα, whereas the BLT1 inhibitor U75302 had no effect (Fig. 5B and C). Treatment with MK886 or baicalein also inhibited the LPS-induced phosphorylation of IκBα (Fig. 5D). We also examined the effects of BLT2 inhibition on LPS-induced nuclear translocation of the NF-κB subunit p65 (Fig. 5E). Immunofluorescence staining revealed a diffuse cytoplasmic localization of p65 in control cells. A pronounced nuclear localization was apparent in cells stimulated with LPS, and this localization was strongly inhibited by LY255283. These data suggest that NF-κB lies downstream of BLT2 in the cascade leading to LPS-enhanced invasiveness in MDA-MB-231 cells.

### BLT2 inhibition significantly reduces LPS-induced metastasis in an orthotopic breast cancer model

**Figure 6:**
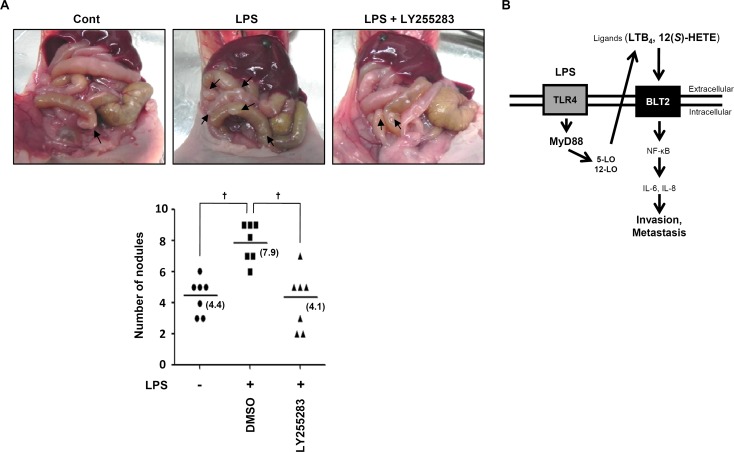
BLT2 inhibition significantly reduces LPS-induced metastasis in an orthotopic breast cancer model (A) MDA-MB-231 cells were incubated for 30 min with LY255283 (10 μM) or DMSO and then for 24 h in the presence or absence of LPS (1 μg/ml), after which, they were implanted into the mammary fat pads of nude mice. Then, the animals were then injected intraperitoneally with LY255283 (2.5 mg/kg) or DMSO three times at 5-day intervals. The mice were killed 14 weeks after implantation, and the development of metastatic nodules (arrows) in the small bowel was examined. The number of nodules per mouse is shown in the bottom panel. Data are representative of results obtained with three mice per group. ^†^*p* < 0.01. (B) Scheme for the involvement of the MyD88-BLT2 cascade in LPS-induced invasiveness of breast cancer cells.

Cell invasiveness is closely associated with metastatic potential [29]. We examined the involvement of BLT2 in LPS-driven metastasis of MDA-MB-231 cells *in vivo*. MDA-MB-231 cells were pretreated with LY255283 (10 μM) or DMSO, followed by LPS (1 μg/ml) for 24 h, before being implanted into the mammary fat pads of mice. 14 weeks after implantation, the mice were killed, and metastatic nodules were counted. The number of nodules in the small bowel was markedly increased by LPS administration and significantly reduced by LY255283 (Fig. 6A). Together, these results indicate that BLT2 plays a crucial role in LPS-induced metastasis in mammary fat pad experiments.

## DISCUSSION

In the present study, we showed that LPS markedly increased BLT2 expression in the highly aggressive breast cancer cells MDA-MB-231 and MDA-MB-435. Selective depletion of BLT2 by RNAi-mediated knockdown resulted in pronounced attenuation of LPS-driven invasion in MDA-MB-231 cells. In addition, we identified MyD88 as being upstream of BLT2 and NF-κB and IL-6/IL-8 as being downstream of BLT2 in the LPS-signaling pathway responsible for stimulating cell invasion. Finally, we showed that LPS-induced metastasis by MDA-MB-231 cells was greatly suppressed by BLT2 inhibition in an orthotopic metastasis model. Together, our findings show that LPS potentiates the invasiveness of aggressive breast cancer cells through a ‘MyD88-BLT2-NF-κB-IL-6/IL-8’ signaling cascade.

Recently, cancers have been reported to become more invasive and aggressive following exposure to LPS via TLR4-MyD88 signaling pathway. For example, LPS has been shown to potentiate the invasiveness and metastasis of cancer cells [8, 9, 25]. In addition, LPS treatment was shown to increase the levels of IL-6 and IL-8, which are closely associated with the invasiveness of breast cancer cells [19, 26]. Similarly, MyD88 has previously been closely related to the occurrence, development, drug resistance and metastasis of cancer [4, 5, 30-36]. In addition, the MyD88-dependent pathway of LPS signaling has been suggested to play a potential prognostic role for tumor malignancy in colorectal cancer development [37], and forced MyD88 expression has been reported to induce the production of inflammatory cytokines such as IL-6 and IL-8 [33]. However, the detailed signaling mechanism involved in ‘LPS-MyD88’-potentiated invasiveness and IL-6/IL-8 production has not been elucidated. Here, we demonstrated that BLT2 is a critical downstream component of LPS-MyD88 signaling that mediates the enhanced invasion of breast cancer cells.

BLT2 is a G-protein-coupled receptor that is expressed on the cell surface and interacts with the specific ligands LTB_4_ and 12(*S*)-HETE. A number of proinflammatory functions of the closely related receptor BLT1 have been characterized, but few biological functions of BLT2 have been identified. However, recent studies have suggested that BLT2 plays a role in cancer progression [12-15, 20, 23]. Previously, BLT2 has been shown to be increased in ovarian, bladder, and prostate cancers, and elevated BLT2 levels have been associated with cancer cell survival, chemoresistance and metastasis [20, 21, 38, 39]. In the present study, we found that LPS upregulated BLT2 in aggressive breast cancer cells (Fig. 1) and that depletion of BLT2 attenuated the ability of LPS to stimulate invasiveness and biosynthesis of IL-6 and IL-8 in these cells (Fig. 2). In addition, our results suggest that 5-LO, 12-LO and their metabolites LTB_4_ and 12(*S*)-HETE are all increased by LPS treatment (Fig. 3A and B). Furthermore, the 5-LO inhibitor MK886 and 12-LO inhibitor baicalein attenuated the LPS-induced invasiveness of breast cancer cells (Fig. 3C). Therefore, LTB_4_ and 12(*S*)-HETE synthesized in response to 5-LO and 12-LO, respectively, likely act via BLT2 in an autocrine or paracrine manner to stimulate the LPS-induced invasiveness of breast cancer cells. Furthermore, our results clearly show that MyD88 acts through BLT2 in MDA-MB-231 cells (Fig. 4) and that the LPS-induced increases in 5-LO and 12-LO and the biosynthesis of their metabolites occurred through MyD88 (Fig. 4C and D). Finally, MyD88 overexpression alone upregulated the levels of 5-LO and 12-LO, as well as the quantity of BLT2 ligands (Fig. 4E and F), indicating that the MyD88-5-LO/12-LO-LTB_4_/12(*S*)-HETE-linked pathway lies upstream of BLT2 in the LPS-potentiated invasiveness in breast cancer cells (as summarized in Fig. 6B).

Our results implicate NF-κB as a key downstream component of the LPS-MyD88-BLT2 pathway. Similar to our results, constitutive NF-κB activation has been observed in most breast cancer cells [40, 41], and the activation of NF-κB is further elevated by LPS-MyD88 signaling [5, 42, 43]. NF-κB activation is believed to increase the synthesis of inflammatory cytokines, such as IL-6 and IL-8. We previously showed that NF-κB is stimulated via a BLT2-linked pathway to promote the invasiveness of breast cancer cells [19], and we demonstrated in the present study that LPS-induced NF-κB activation is critical for invasive potential (Fig. 5A). Furthermore, LPS-induced NF-κB activation was reduced by BLT2 inhibition with siRNA or LY255283 (Fig. 5B, C and E). Thus, we propose that an LPS-MyD88-BLT2 signaling cascade regulates NF-κB activation in breast cancer cells.

Breast cancer cells are metastasized to distant organs, preferentially, to the lung, liver and bones. In addition, metastasis to the gastrointestinal has observed 16 % cases in the case reports of breast cancer patients [44-48]. Interestingly, our *in vivo* LPS-driven orthotopic breast cancer model reproducibly shows the metastasis to the gastrointestinal organ, small bowel (Fig. 6). In any event, our results show that LPS-enhanced metastasis to small bowel was remarkably reduced by treatment of BLT2 inhibitor LY255283 (Fig. 6A), suggesting that BLT2 might be associated with the LPS-induced breast cancer metastasis. Further studies are needed to investigate the exact role of BLT2 for breast cancer metastasis in response to LPS exposure.

In summary, we showed that LPS potentiates the invasiveness of aggressive breast cancer cells through a ‘MyD88-BLT2-NF-κB-IL-6/IL-8’ signaling cascade. The elucidation of this mechanism provides important insights into breast cancer progression, especially in inflammatory condition.

## MATERIALS AND METHODS

### Materials

Fetal bovine serum (FBS) and RPMI 1640 were obtained from Life Technologies (Gaithersburg, MD), and MK886, baicalein, U75302 and LY255283 were acquired from Cayman Chemical Co. (Ann Arbor, MI). LPS (*Escherichia coli* serotype O55:B5), bovine serum albumin, and dimethyl sulfoxide (DMSO) were acquried from Sigma-Aldrich (St. Louis, MO), and Bay11-7082 was from Calbiochem (La Jolla, CA). Antibodies to 5-lipoxygenase, 12-lipoxygenase and p65 were obtained from Santa Cruz Biotechnology (Santa Cruz, CA), and antibodies to phospho-IκBα, β-actin and α-tubulin were from Cell Signaling Technology (Danvers, MA). All other chemicals were obtained from standard sources and were of molecular biology grade or higher.

### Cell culture

The human breast cancer cell lines MDA-MB-231 and MDA-MB-435 were obtained from the Korean Cell Line Bank (Seoul, Korea) and Jackson Laboratory (Bar Harbor, ME), respectively. These cells were maintained in RPMI-1640 containing 10% heat-inactivated FBS and antibiotic-antimycotic solution (Life Technologies, Gaithersburg, MD) at 37 °C in a humidified atmosphere of 5% CO_2_. Cell lines were routinely authenticated through cell morphology monitoring, growth curve analysis and identity verification using mycoplasma detection kit (Lonza, Rockland, ME) to ensure no contamination. Cells were last tested a week before all experiments were performed.

### Semiquantitative RT-PCR and quantitative real-time PCR analysis

Total RNA was extracted from cells with Easy-Blue (Intron, Sungnam, Korea), and a portion (1.25 μg) of the RNA was subjected to reverse transcription (RT) with M-MLV reverse transcriptase (Beams Bio, Gyunggi, Korea), followed by semiquantitative PCR analysis with a PCR PreMix Kit (Intron) under conditions optimal for linear amplification of GAPDH cDNA. The primer sequences used were as follows: human BLT1 (forward, 5′-TATGTCTGCGGAGTCAGCATGTACGC-3′; reverse, 5′-CCTGTAGCCGACGCCCTATGTCCG-3′) [18]; human BLT2 (forward, 5′-AGCCTGGAGACTCTGACCGCTTTCG-3′; reverse, 5′-GACGTAGCACCGGGTTGACGCTA-3′) [18]; GAPDH (forward, 5′-CTGCACCACCAACTGCTTAGC-3′; reverse, 5′-CTTCACCACCTTCTTGATGTC-3′) [18]; human IL-6 (forward, 5′-CCAGTACCCCCAGGAGAAGA-3′; reverse, 5′-GCATCCATCTTTTTCAGCCA-3′) [22]; human IL-8 (forward, 5′-ATGACTTCCAAGCTGGCCGTGGCT-3′; reverse, 5′-TCTCAGCCCTCTTCAAAAACTTCTC-3′) [23]; and human MyD88 (forward, 5′-TCTCTGTTCTTGAACGTGCGGACA-3′; reverse, 5′-TTTGGCAATCCTCCTCAATGCTGG-3′). The specificity of all primers was confirmed by sequencing the PCR products. For real-time quantitative PCR analysis, BLT1, BLT2, and GAPDH cDNAs were amplified as described previously [15] with a LightCycler 480 SYBR Green I Master kit (Roche Diagnostics, Mannheim, Germany). All data were normalized by GAPDH. The primer sequences used for BLT1 and BLT2 were (forward, 5′-CCTGAAAAGGATGCAGAAGC-3′; reverse, 5′-AAAAAGGGAGCAGTGAGCAA-3′) and (forward, 5′-CTTCTCATCGGGCATCACAG-3′; reverse, 5′-TCCTTCTGGGCCTACAGGT-3′), respectively.

### Immunoblot analysis

The cells were washed with ice-cold PBS, scraped into a lysis buffer (20 mM Tris-HCl, pH 7.5, 150 mM NaCl, 0.5 % Nonidet P-40, 5 mM EDTA, 1 % Triton X-100, and protease inhibitors (100 mM phenylmethylsulfonyl fluoride, 1 mM sodium orthovanadate, 2 μg/ml leupeptin, and 2 μg/ml aprotinin)) at 4 °C, and heated at 95 °C for 5 min. The samples were then subjected to SDS-PAGE, and the separated proteins were transferred electrophoretically to a PVDF membrane for 90 min at 100 V. The membrane was exposed for 1 h to TBST containing 0.05 % Tween 20 and 5 % dried nonfat milk before incubation overnight at 4 °C with antibodies. Antibodies to 5-lipoxygenase, 12-lipoxygenase and p65 were obtained from Santa Cruz Biotechnology (Santa Cruz, CA), and antibodies to phospho-IκBα, β-actin and α-tubulin were from Cell Signaling Technology (Danvers, MA). Blots were developed with a peroxidase-conjugated secondary antibody, and protein were visualized using ECL reagents (Amersham, Arlington Heights, IL) according to the manufacturer's recommendations.

### Invasion assay

The invasive potential of MDA-MB-231 and MDA-MB-435 cells was assessed using BioCoat Matrigel Invasion Chambers (BD Biosciences, Bedford, MA) as described [19]. Cells (3.5 × 10^4^) were harvested with RPMI 1640 supplemented with 0.5% FBS and seeded in the same media on rehydrated Matrigel inserts. RPMI 1640 supplemented with 5% FBS and then added to the lower chamber as a chemoattractant. MDA-MB-231 cells were incubated at 37 °C for 24 h and MDA-MB-435 cells were incubated at 37 °C for 48 h. The cells on the upper surface of each filter were removed, and the remaining cells were fixed in methanol, stained with hematoxylin-eosin (H&E) and counted in 10 randomly selected high-power (40X) fields with a CKX41 microscope (Olympus, Tokyo, Japan) equipped with a DP71 digital camera (Olympus). Each sample was assayed in triplicate.

### RNA interference (RNAi) of BLT2 and MyD88

BLT2-specific(5′CCACGCAGUCAACCUUCUG-3′) [15], MyD88-specific (5′-AGUAGAGCACAGAUUCCUC-3′; No. 1100256) and control (scrambled) siRNAs were obtained from Bioneer (Daejeon, Korea). The siRNAs were introduced into cells by transfection for the indicated times in Opti-MEM (Invitrogen, Carlsbad, CA) with the use of Oligofectamine reagent (Invitrogen).

### Forced expression of MyD88

Cells were transiently transfected with 1 μg of expression vectors for human MyD88 (pCMV-Flag-MyD88, kindly provided by Dr. T. Renno) [24] or the corresponding empty vector (pCMV-Flag) using Lipofectamine reagent (Invitrogen).

### Immunofluorescence staining of p65

Cells were washed twice with ice-cold PBS, fixed with 4% paraformaldehyde, permeabilized with 0.1% Triton X-100, and incubated with antibodies to p65 at a dilution of 1:100 and then with fluorescein isothiocyanate-conjugated secondary antibodies (Molecular Probes) at a dilution of 1:200 as described previously [20]. Each step was performed in PBS containing 1% bovine serum albumin, and the cells were washed between steps with PBS three times for 5 min. Nuclei were stained with 4′,6-diamidino-2-phenylindole (DAPI) (Sigma). Coverslips were mounted on slides with 50% glycerine, and the cells were examined with a confocal laser-scanning microscope (LSM 700; Carl Zeiss, Oberkochen, Germany).

### ELISAs

Enzyme-linked immunosorbent assay (ELISA) kits for human IL-6 and IL-8 were obtained from AbFrontier (Seoul, Korea) and BD Biosciences (Bedford, MA). ELISA kits for human LTB_4_ and 12(*S*)-Hydroxyeicosatetraenoic Acid (12(*S*)-HETE) were obtained from Enzo life sciences (Farmingdale, NY).

### *In vivo* metastasis assays

The study was conducted in strict accordance with the recommendations in the Guide for the Care and Use of Laboratory Animals of Korea University, and the protocol was approved by the Committee on the Ethics of Animal Experiments of Korea University (Permit Number: KUIACUC-2014-103). All animals were housed at a 12:12-h light:dark ratio at a density of 7 mice per static polycarbonate microisolator cage on disposable bedding. Wire-lid food hoppers within cages were filled to capacity with rodent chow, and the mice were maintained with water supplied by a bottle. For spontaneous metastasis assays, cultured MDA-MB-231 cells were pretreated with LY255283 or DMSO, followed by LPS (1 μg/ml) for 24 h, as described previously [19]. Then, six-week-old female nude (BALB/C) mice (Charles River, Wilmington, MA) were injected unilaterally into the fourth right mammary fat pad with cultured MDA-MB-231 (2.0 × 10^6^) cells in 100 μl of PBS by subcutaneous injection at the base of the nipple. LY255283 (2.5 mg/kg) or DMSO vehicle was injected intraperitoneally three times at 5-day intervals beginning immediately after cell implantation. Animals were sacrificed 14 weeks after injection, and the number of metastatic nodules on the surface of the small bowel was determined.

### Data analysis and statistics

The data are representative of three independent experiments. The results are presented as the mean ± standard deviation (SD). Comparisons between groups were performed with Student's *t*-test using SigmaPlot 8.0 software. *P*-values less than 0.05 were considered significant.

## Abbreviations

LPS: lipopolysaccharide
TLR4: Toll-like receptor 4
MyD88: Myeloid differentiation primary response gene 88
LTB4: leukotriene B4
12(S)-HETE: 12(S)-Hydroxyeicosatetraenoic Acid
5-LO: 5-lipoxygenase
12-LO: 12-lipoxygenase
